# 

**Published:** 1898-08

**Authors:** 


### CONTRIBUTORS TO VOLUME XXXVIII.

**New Series—August, 1898-July, 1899.**

---

| | |
|---|---|
| BABCOCK, C. E. P. | Buffalo. |
| BENEDICT, A. L. | Buffalo. |
| BISHOP, EDWIN R. | Geneva. |
| CLARK, EDWARD | Buffalo. |
| CLEMESHA, J. C. | Buffalo. |
| CLINTON, MARSHALL | Buffalo. |
| DAGGETT, B. H. | Buffalo. |
| DOOLEY, E. M. | Buffalo. |
| DOWD, J. HENRY | Buffalo. |
| DUNHAM, SYDNEY A. | Buffalo. |
| DURAND, CHARLES F. | Buffalo. |
| FREDERICK, C. C. | Buffalo. |
| FRONCZAK, FRANCIS E. | Buffalo. |
| FRYE, MAUD J. | Buffalo. |
| GRANT, JOHN H. | Buffalo. |
| GREENE, WALTER D. | Buffalo. |
| HARRIS, PETER C | U. S. Army. |
| HARTWIG, MARCELL | Buffalo. |
| HEATH, WILLIAM H. | Buffalo. |
| HIGGINS, F. W. | Cortland. |
| HIMMELSBACH, G. A. | Buffalo. |
| HOWE, LUCIEN | Buffalo. |
| HUBBELL, ALVIN A. | Buffalo. |
| JACK, GEORGE N. | Depew. |
| JAMESON, THOMAS | Rochester. |
| JONES, WILLIAM B | Rochester. |
| KERR, ABRAM T., JR. | Buffalo. |
| KING, CLARENCE | Machias. |
| KNAPP, LOUIS H. | Buffalo. |
| KRAUSS, WILLIAM C. | Buffalo. |
| LOOP, GEORGE ROSS | Buffalo. |
| LOVELAND, B. C. | Clifton Springs. |
| LUSK, ZERA J. | Warsaw. |
| LYTLE, ALBERT T. | Buffalo. |
| MANN, MATTHEW D. | Buffalo. |
| MILLENER, FREDERICK H. | Buffalo. |
| MILLER, JOHN A. | Buffalo. |
| PARK, ROSWELL | Buffalo. |

Parmenter, John . . . . . . . . . . . . . . . . . . . . . . . . . Buffalo.
Pierce, L. Clark . . . . . . . . . . . . . . . . . . . . . . . . . Sonyea.
Potter, William Warren . . . . . . . . . . . . . . . . . . . . Buffalo.
Price, Joseph . . . . . . . . . . . . . . . . . . . . . . . . Philadelphia.
Pryor, John H. . . . . . . . . . . . . . . . . . . . . . . . . . Buffalo.
Putnam, James Wright . . . . . . . . . . . . . . . . . . . . . . Buffalo.
Roe, John O. . . . . . . . . . . . . . . . . . . . . . . . . . Rochester.
Rogers, Benjamin F. . . . . . . . . . . . . . . . . . . . . . . Buffalo.
Satterlee, Richard H. . . . . . . . . . . . . . . . . . . . . . Buffalo.
Spangenthal, Joseph . . . . . . . . . . . . . . . . . . . . . . Buffalo.
Stockton, Charles G. . . . . . . . . . . . . . . . . . . . . . Buffalo.
Tait, Lawson . . . . . . . . . . . . . . . . . . . . . Birmingham, England.
Taylor, William G. . . . . . . . . . . . . . . . . . . . . . . Buffalo.
Trowbridge, Grosvenor R. . . . . . . . . . . . . . . . . . . . Buffalo.
Uhlman, Julius . . . . . . . . . . . . . . . . . . . . . . . . Buffalo.
Van de Warker, Ely . . . . . . . . . . . . . . . . . . . . . . Syracuse.
Veeder, M. A. . . . . . . . . . . . . . . . . . . . . . . . . . Lyons.
Warren, William M. . . . . . . . . . . . . . . . . . . . . . . Detroit.
Weigel, L. A. . . . . . . . . . . . . . . . . . . . . . . . . Rochester.
Wende, Ernest . . . . . . . . . . . . . . . . . . . . . . . . . Buffalo.
Wende, Grover William . . . . . . . . . . . . . . . . . . . . . Buffalo.
Williams, H. T. . . . . . . . . . . . . . . . . . . . . . . . Rochester.
Wilson, Nelson W. . . . . . . . . . . . . . . . . . . . . . . . Buffalo.
Young, A. A. . . . . . . . . . . . . . . . . . . . . . . . . . . Newark.
Buffalo Academy of Medicine.
Buffalo Sanatory Club.
Medical Association of Central New York.
Medical Society of the County of Erie.

logical order from 1565 to 1898. A well-arranged index closes one of the best essays on the subject that we have lately seen and we hope the author's implied promise of successful treatment will be realised for the sake of both physician and patient.

---

### BOOKS RECEIVED.

System of Diseases of the Eye. By American, British, Dutch, French, German and Spanish Authors. Edited by William F. Norris, A. M., M. D., and Charles A. Oliver, A. M., M. D., of Philadelphia. Vol. III. Local Diseases, Glaucoma, Wounds and Injuries, Operations. Imperial octavo, pp. xii.—962. With 50 full-page plates and 186 text illustrations. Philadelphia: J. B. Lippincott Company. 1898.

A Manual of Modern Surgery, General and Operative. By John Chalmers DaCosta, M. D., Clinical Professor of Surgery, Jefferson Medical College, Philadelphia, etc. Octavo, pp. 911. With 386 illustrations. Price, cloth, $4.00; half morocco, $5.00 net. Philadelphia: W. B. Saunders, 925 Walnut street. 1898.

Electricity in the Diagnosis and Treatment of Diseases of the Nose, Throat, and Ear. By W. Scheppegrell, A. M., M D., ex-Vice-president American Laryngological, Rhinological and Otological Society, etc., etc. Octavo, pp. xiv.—403. New York: G. P. Putnam's Sons, 27 West Twenty-third street. 1898.

A System of Practical Medicine. By American Authors. Edited by Alfred Lee Loomis, M. D., late Professor of Pathology and Practical Medicine in the New York University, and William Gilman Thompson, M. D., Professor of Medicine in the Cornell University Medical College, New York. In four imperial octavo volumes. Vol. IV.—Diseases of the Nervous System and Mind; Vasomotor and Trophic Disorders; Diseases of the Muscles; Osteo-Malacia; Rachitis; Rheumatism; Arthritis; Gout; Lithemia; Obesity; Scurvy; Addison's Disease. 1,099 pages, 95 engravings, and 8 full-page plates in colors and monochrome. For sale by subscription. Per volume, cloth, $5.00; leather, $6.00; half morocco, $7.00. Lea Brothers & Co., Publishers, Philadelphia and New York. 1898.

International Clinics. A Quarterly of Clinical Lectures and Specially Prepared Articles on Treatment and Drugs. By professors and lecturers in the leading medical colleges of the United States, Germany, Austria, France, Great Britain and Canada. Edited by Judson Daland, M. D. (Univ. of Penna.), Philadelphia, Instructor in Clinical Medicine and Lecturer on Physical Diagnosis in the University of Pennsylvania; Assistant Physician to the Hospital of the University of Pennsylvania, etc.; Fellow of the College of Physicians of Philadelphia. J. Mitchell Bruce, M. D., F. R. C. P., London, England, Physician to and Lecturer on the Principles and Practice of Medicine at the Charing Cross Hospital. David W. Finlay, M. D., F. R. C. P., Aberdeen, Scotland, Professor of Practice of Medicine in the University of Aberdeen; Physician to and Lecturer on Clinical Medicine in the Aberdeen Royal Infirmary, etc. Volume II. Eighth series. 1898. Octavo, pp. xii.—366. Philadeldhia: J. B. Lippincott Co. 1898.

Manual of Practical Diagnosis for the use of Students and Physicians. By James Tyson, M. D., Professor of Clinical Medicine in the University of Pennsylvania, and Physician to the University Hospital, etc. Small Duodecimo, pp. xii.—278. Third edition, revised and enlarged. Illustrated. Price, $1.50 net. Philadelphia: P. Blakiston, Son & Co., 1012 Walnut street. 1898.

Atlas and Epitome of Operative Surgery. By Dr. Otto Zuckerkandi, Privatedocent in the University of Vienna. Authorised translation from the German. Edited by J. Chalmers DaCosta, M. D., Clinical Professor of Surgery in Jefferson Medical College, Philadelphia, etc. Duodecimo, pp. 395. With 24 colored plates and 217 illustrations in the text. Price, $3.00 net. Philadelphia: W. B. Saunders. 1898.

Atlas of Syphilis and the Venereal Diseases, including a brief treatise on the Pathology and Treatment. By Prof. Dr. Franz Mracek, of Vienna. Authorised translation from the German. Edited by L. Bolton Bangs, M. D., Consulting Surgeon to St. Luke's Hospital and the City Hospital, New York, etc., etc. Duodecimo, pp. 122. With 71 colored plates. Price, $3.50 net. Philadelphia: W. B. Saunders. 1898.

Diseases of the Nervous System. A hand-book for Students and Practitioners. By Charles E. Beevor, M. D., London, F. R. C. P. Physician to the National Hospital for the Paralysed and Epileptic, etc. Duodecimo, pp. xv.—432. Illustrated. Price, $2.50 net. Philadelphia: P. Blakiston, Son & Co., 1012 Walnut street. 1898.

A Compend of Diseases of the Skin. By Jay F. Schamberg, A. B., M. D., Associate in Skin Diseases, Philadelphia Polyclinic, etc., etc. Illustrated. Price, 80 cents. Philadelphia: P. Blakiston, Son & Co. 1898.

Retinoscopy (or shadow test) in the Determination of Refraction at one Meter Distance, with the Plane Mirror. By James Thorington, M. D., Adjunct Professor of Diseases of the Eye in the Philadelphia Polyclinic and College for Graduates in Medicine, etc., etc. Second edition, revised and enlarged. Illustrated. Philadelphia: P. Blakiston, Son & Co. 1898.

Twenty-second Year Book of the New York State Reformatory. For the fiscal year ending September 30, 1897. With illustrations and tables. Elmira, N. Y. 1898.

Report of the Commissioner of Education for the Year 1896-97. Volume I. Containing Part I. Washington: Government Printing Office. 1898.

Twenty-fourth Annual Report of the Secretary of the State Board of Health of the State of Michigan for the Fiscal Year Ending June 30, 1898. Lansing. 1897.

Seventeenth Annual Report of the State Board of Health of New York. Transmitted to the legislature February 15, 1897. New York and Albany. 1898.

The Johns Hopkins Hospital Reports. Report in Pathology. Volume VII., No. 3. Baltimore: The Johns Hopkins Press. 1898.

The Office Treatment of Hemorrhoids, Fistula, etc., without Operation. Together with remarks on the relation of diseases of the rectum to other diseases in both sexes, but especially in women, and the abuse of the operation of colostomy. By Charles B. Kelsey, A. M., M. D., late Professor of Surgery at the New York Post-graduate Medical School and Hospital; Fellow of the New York Academy of Medicine, the New York County Medical Society, etc. Duodecimo, pp. 68. Price, 75 cents, net. New York: E. R. Pelton, Publisher, No. 19 East Sixteenth street. 1898.

